# Water as a Sustainable Leaching Agent for the Selective Leaching of Lithium from Spent Lithium-Ion Batteries

**DOI:** 10.1021/acsomega.3c07405

**Published:** 2024-02-09

**Authors:** Rafaela Greil, Joevy Chai, Georg Rudelstorfer, Stefan Mitsche, Susanne Lux

**Affiliations:** † Institute of Chemical Engineering and Environmental Technology, Graz University of Technology, NAWI Graz, Inffeldgasse 25C, Graz 8010, Austria; ‡ Chemical Engineering Department, Universiti Teknologi PETRONAS, Seri Iskandar 32610, Malaysia; § Institute for Electron Microscopy and Nanoanalysis and Center for Electron Microscopy, Graz University of Technology, NAWI Graz, Steyrergasse 17, Graz 8010, Austria

## Abstract

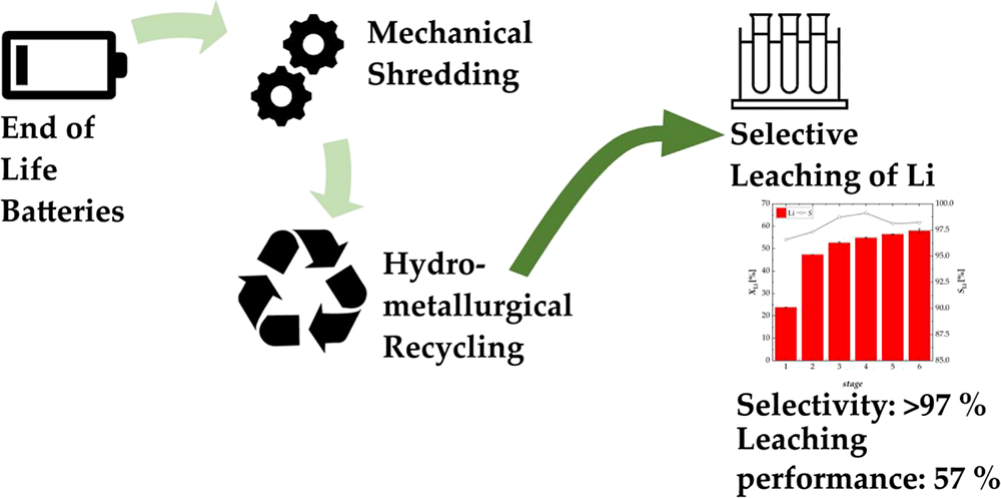

The development of a sustainable recycling process for lithium from spent lithium-ion batteries is an essential step to reduce the environmental impact of batteries. So far, the industrial implementation of a recycling process for lithium has been hindered by low recycling efficiencies and impurities in the recycled material. The aim of this study is thus to develop an easy-to-implement recycling concept for the selective leaching of lithium from spent lithium-ion batteries with water as a sustainable leaching reagent. With this highly selective process, the quantity of chemicals used can be substantially decreased. The influence of the leaching temperature, the solid/liquid-ratio, the mixing rate, and the number of stages in multistage operation were investigated utilizing NCM-material. High leaching efficiencies and a high selectivity were achieved at moderate temperatures of 40 °C and a solid/liquid-ratio of 100 g L^–1^. In multistage operation, a selectivity for lithium higher than 98% was achieved with 57% leaching performance of lithium. XRD-measurements showed that lithium carbonate was quantitatively leached, while lithium metal oxides remained in the black mass. Finally, the leaching kinetics were determined, proving that the first leaching period is diffusion controlled and, in the second period, the leaching rate is rate controlling. This work confirms the concept of a green leaching process by which lithium can be recycled with a high degree of purity.

## Introduction

The breakthrough of electric mobility and portable electrical devices would not have been possible without lithium-ion batteries (LIBs). Lithium, as one of the key elements of the active cathode material, is obtained by extraction from either salt lakes or ores. The extraction from salt lakes requires large quantities of water and is time-consuming (approximately 18 months). The extraction from ores is an expensive and energy-intensive process. Moreover, if lithium is not recovered by recycling, it has a forecasted shortage of almost 47 million tons by 2050.^1−4^ In the face of the growing scarcity of raw materials and the ever-increasing demand for lithium-ion batteries, the development of a recycling concept for spent LIBs has become increasingly important in recent years.^1^ The main focus is usually put on recycling of the cathode material, as it accounts for 44% of the material costs of a LIB.^5^ In principle, the cathode is manufactured by using an active material (e.g., LCO: LiCoO_2_, LFP: LiFePO_4_, LMO: LiMn_2_O_4_, NCA: LiAl_*x*_Co_*y*_Ni_1–*x*_–_*y*_O_2_, and NCM: LiCo_*x*_Mn_*y*_Ni_1_–_*x*_–_*y*_O_2_), a collector foil (aluminum or copper), and a binder (e.g., polyvinylidene fluoride). In previous years, the NCM material has been mostly applied as the active material, owing to its high energy density (592–740 W h kg^–1^) paired with a low price (145–230 US$ kW h^–1^).^6^

Several process combinations have been investigated for the recycling of LIBs. These consist of discharging, mechanical and/or thermal pretreatment, and/or pyrometallurgical and/or hydrometallurgical recycling technologies.^2^ Discharging is accomplished using salt-water baths (e.g., NaCl), thermal processing, or controlled discharging via external circuits. Mechanical pretreatment is executed to remove the casing and foils from the active material (mixture of the cathode and anode material) using a combined multistage process of crushing, sieving, magnetic separation, fine crushing, and classification. Thermal pretreatment is often applied to remove the electrolyte and the binder, which results in a more efficient hydrometallurgical recycling. The result is a black powder containing the active material, which is known as black mass.^7−10^ Pyrometallurgical recycling combines smelting and roasting steps to produce battery slag, from which nickel, cobalt, and copper can be recovered. The main disadvantages are the emission of hazardous gases, the need of purification steps for recycling of the valuable metals (e.g., hydrometallurgical processing), and the fact that, until today, the recovery of lithium has been lower than 10%. Moreover, a high input of electrical energy (4.68 MJ kg_battery_^–1^) is required.^11^ Hydrometallurgical recycling comprises leaching, meaning the dissolution of metals from the black mass plus purification steps, which include ion exchange, precipitation, and liquid–liquid extraction. These process steps require less electrical energy (0.125 MJ kg_battery_^–1^) compared to pyrometallurgical recycling. For leaching, the usage of mineral acids (e.g., sulfuric, hydrochloric and nitric acid) and organic acids (e.g., citric, oxalic, and malic acid) in combination with or without a reducing agent (e.g., hydrogen peroxide) are primarily studied.^7,8,12−15^ After leaching, various extraction and precipitation steps follow in order to recover the valuable metals from the leachate. Neumann et al., for instance, presented a traditional hydrometallurgical process, where after leaching, first, precipitation of impurities (Al, Fe, Cu), second, a multistage solvent extraction followed by precipitation of manganese, cobalt, and nickel, and finally, precipitation of lithium took place. In this study, a significant loss of lithium was reported because of its low concentration in the leachate (∼10 g L^–1^). Furthermore, impurities may be present in the solid product from lithium precipitation due to residual metal concentrations rendering the precipitate unusable for battery production.^8^ Lithium is typically recovered as lithium carbonate via homogeneous (with Na_2_CO_3_ as the precipitation aid) or heterogeneous (with CO_2_ as the precipitation aid) precipitation. Zhu et al. proposed leaching of the LiCoO_2_ material with 2 M H_2_SO_4_ and 2 wt % H_2_O_2_ followed by precipitation of cobalt with ammonium oxalate in 1.2 molar excess. They finally precipitated lithium using sodium carbonate in 1.1 molar excess, at an equilibrium pH of 10, 50 °C, and 1 h reaction time. They showed that a lithium concentration of 20 g L^–1^ gives the highest precipitation efficiency (>80%).^16^ It is evident that a selective leaching process with a simple precipitation step is essential for the direct recovery of lithium carbonate with high efficiency and purity.^2,5,6,8^

In recent years, classical leaching methods with acids (e.g., formic acid) together with new approaches using a strong oxidizing environment, supercritical CO_2,_ or water combined with thermal pretreatment have been explored. Acids are most commonly used as leaching reagents. For instance, formic acid was utilized to selectively leach lithium from various NCM-materials. With concentrated formic acid at 60 °C and 5 h reaction time, a leaching efficiency of 100% of lithium was achieved with less than 5% leaching efficiency of other metals.^17^ Nevertheless, formic acid is difficult to handle in industrial applications because it burns in the presence of oxygen to form carbon dioxide and water. In contrast, Lv et al. investigated an advanced oxidation process for a NCM-material using sodium persulfate as a leaching reagent. They showed that 91.23% of lithium together with 17.56% of nickel were leached at 85 °C and a solid/liquid (S/L)-ratio of 400 g L^–1^. Supercritical CO_2_ was also utilized to selectively leach lithium from the black mass. Swich et al. and Pavón et al. showed a leaching process with supercritical CO_2_ and water using the NCM-material at 230 °C and a S/L-ratio of 30–100 g L^–1^ with a maximum lithium yield of 94.5%.^9,18^ Recently, water as a leaching agent has been proposed in combination with various pretreatment methods, such as reduction/roasting methods (e.g., carbothermic reduction, oxidation, and vacuum pyrolysis) and nitration methods. Carbothermic reduction was operated at temperatures of 500–800 °C under inert (e.g., argon, nitrogen, methane) or reactive atmosphere (e.g., CO_2_).^19−25^ Results with the NCM-material showed that, after carbothermic reduction, nickel and cobalt were present in their metal form or/and as oxide, manganese was present as oxide, and lithium as carbonate and oxide, if the roasting temperature was below 700 °C.^21,24,25^ If LiCoO_2_ material was utilized, lithium was reduced to water-soluble oxide and carbonate and cobalt to water-insoluble metal and oxide.^23^ A leaching efficiency of lithium of almost 60% was achieved when the carbothermally reduced NCM-material was leached with water at 80 °C for 3 h.^25^ Zhu et al. investigated a carbothermal shock method at 2200 °C and 20 s using various types of black mass (NCM111, NCM523, NCM622, NCM811, LiCoO_2,_ and LiMn_2_O_4_), in which lithium was converted to oxide.^26^ Carbothermic oxidation was operated at temperatures of 400–700 °C and times of 30–90 min. Results showed that the carbon content was decreased by 40 wt % and CoO, Co_3_O_4_, NiO, Mn_3_O_4_, MnO_2_, Li_2_O, and Li_2_CO_3_ were formed. The leaching efficiency of lithium was 30% at 80 °C and 3 h.^25,27^ Xiao et al. investigated the vacuum pyrolysis of the mixed cathode powder (LiCoO_2_, LiMn_2_O_4,_ and NCM) at temperatures of 700 °C and 30 min and obtained a leaching efficiency of lithium of 81.9%. Peng et al. investigated a nitration procedure (70 °C, 45% HNO_3_) followed by selective roasting (250 °C) using the NCM-material. They showed that after nitration, lithium was present as nitrate and other metals (e.g., manganese, nickel, cobalt, and copper) as oxides. Furthermore, this process could increase the leaching efficiency of lithium by ca. 10%.^28^ Further research has been conducted using a roasting process with different additives (e.g., (NH_4_)_2_C_2_O_4_, H_2_, and K_2_S_2_O_7_) at 500–700 °C or a mechanochemical process at 25 °C.^29−33^ After the mixture was roasted, selective water leaching was carried out. Leaching efficiencies for lithium of 90–98% were achieved with temperatures of 25–60 °C, S/L-ratios of 5–50 g L^–1^ for LCO, and the NCM-material in a single-stage leaching process.^19−23,26,29,32,33^ Peng et al. investigated a four-stage cross-current leaching process at 25 °C and a S/L-ratio of 500 g L^–1^ with a lithium concentration of 34.2 g L^–1^ in the leaching solution and obtained a leaching efficiency of 93%.^28^ Hu et al. executed carbonated water leaching after carbothermal reduction utilizing the NCM-material at 25 °C, a S/L-ratio of 20 g L^–1,^ and a gas feed stream of 20 mL min^–1^, where more soluble LiHCO_3_ compared to LiMO_2_ (M represents Ni, Mn, Co, and Cu) was formed. The leaching efficiency of lithium was 84.7%.^20^ These studies showed that water-soluble Li_2_CO_3_ or LiHCO_3_ were formed during roasting, which strongly increased the efficiency of the selective leaching process of lithium using water as a leaching reagent. However, thermal pretreatments are energy intensive, and in the case of reductive roasting and nitration, further usage of chemicals is necessary.

In order to reduce both the chemicals and energy requirements while ensuring high selectivity for lithium, the potential of a selective process for leaching of lithium with water as the leaching reagent without any thermal pretreatment of the black mass is investigated in this study. The NCM material was utilized, which, to the best of our knowledge, has not yet been studied. The effect of the operating parameters such as temperature, S/L-ratio, and stirring speed was intensively studied in a single-stage process. Based on the experimental data, kinetic modeling was accomplished. Furthermore, a multistage process was investigated. This concept comprises a highly selective leaching process with low chemical and energy consumption.

## Experimental Section

### Materials and Characterization

The black mass, which is a mixture of various LiCo_*x*_Mn_*y*_Ni_1_–_*x*_–_*y*_O_2_ (NCM), was supplied by Redux GmbH. The supplied material had undergone a partial pretreatment, where organic and electrolyte compounds were separated from the black mass. Deionized water was utilized as a leaching reagent in this study.

The metal concentration of the black mass and in the leaching solution was analyzed by atomic absorption spectroscopy. Multiple samples of the black mass were dissolved in aqua regia (3:1 concentrated HCl:H_2_SO_4_), where 1.5 g of black mass was dissolved in 10 mL of aqua regia at 25 °C to determine the dissolved metal concentration. Samples of the dissolved black mass and the leaching solution were diluted with a HNO_3_ solution (*w* = 0.65%, concentrated HNO_3_ (J.T. Baker, 65%) in ultrapure water). The atomic absorption spectrometer (AAS, PerkinElmer AANALYST 400), was equipped with an autosampler (PerkinElmer AS-90plus), a hollow cathode lamp for lithium (PerkinElmer), and a multicomponent hollow cathode lamp for Co/Cr/Cu/Fe/Mn/Ni (PerkinElmer). The flame was controlled with acetylene and air. The mass fractions (*w*) of each metal are shown in Table 1. The mass fractions of aluminum and carbon were provided by the manufacturer.

**Table 1:** Composition of the NCM-Material Measured with Atomic Absorption Spectroscopy

	Li	Co	Mn	Ni	Cu	Fe	Al^a^	C^a^
*w* [%]	3.95	7.90	5.54	14.46	4.41	1.43	5.73	30.5

a Provided by the manufacturer.

The particle size distribution of the NCM-material was determined by laser diffraction (Sympatec Helios KR with a cuvette (50 mL) and a dispersing system) with a measurement range of 0.45–875 μm. The sample was dispersed in isopropyl alcohol (Roth, ≥99.9%). Prior to the measurement, the sample was exposed to ultrasound for 50 s (stirrer speed: 500 rpm) to prevent agglomeration. Figure S1 in the Supporting Information shows the particle size distribution of the NCM-material with a mean diameter of 14.85 μm.

The density of the NCM-material was measured with a pycnometer (Brand, 100 mL). It is 1575 g L^–1^.

Powder X-ray diffraction (XRD) was applied to determine the phases of the NCM-material and the leached sample 1′ of the multistage process. Samples were analyzed using a Siemens D5005 X-ray diffractometer (Munich, Germany) configured with Bragg–Brentano geometry that implements Cu Kα (1.5418 Å). A current of 40 mA and a voltage of 40 kV were applied. Scans of 4°–90° and 20°–42° 2θ were performed. A step size of 0.04° was utilized for each measurement. The measurement time was 2 s for the long scan and 10 s for the detail scan.

**Figure 1:**
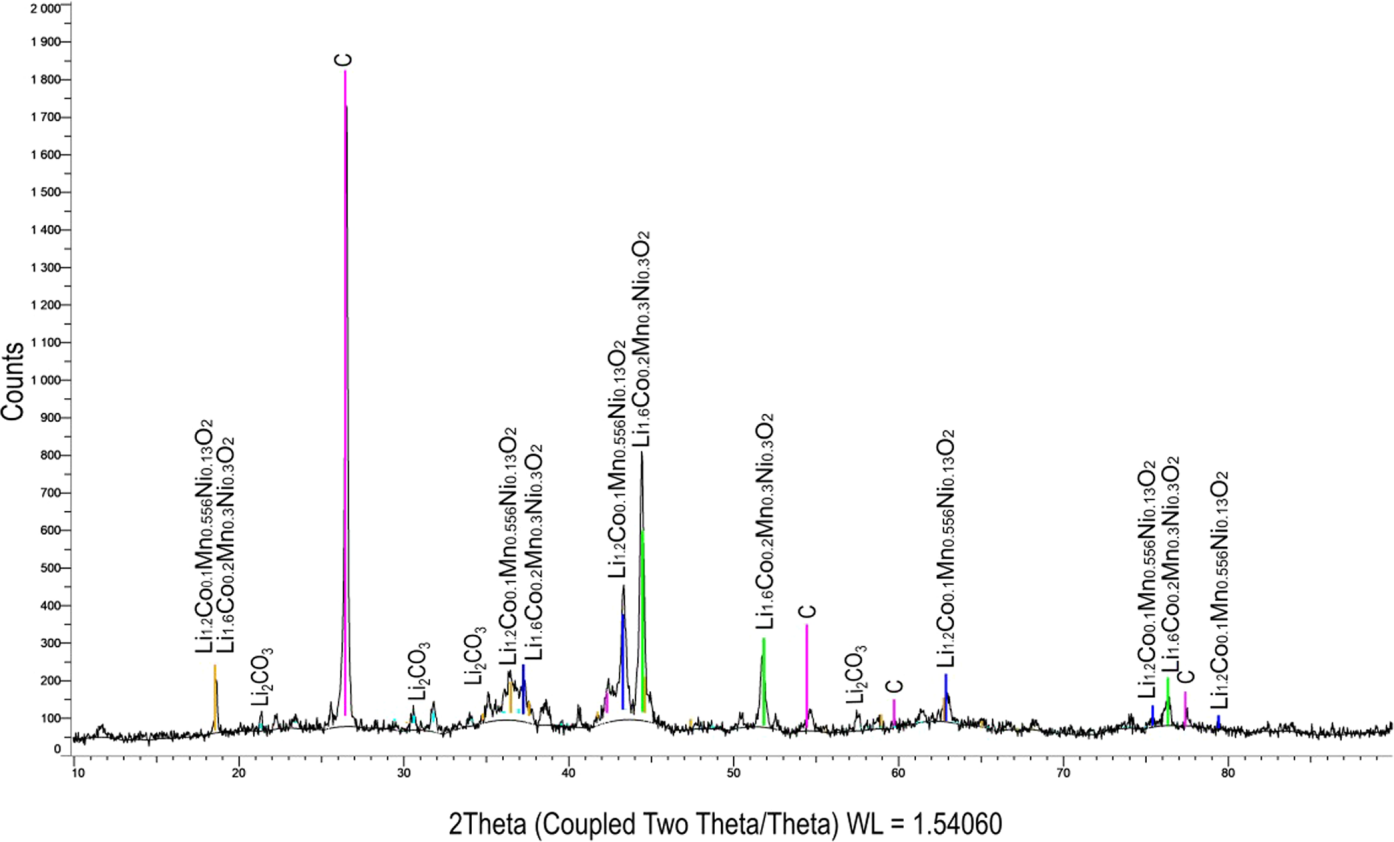
XRD-measurement of the NCM-material (current of 40 mA, voltage of 40 kV, scans of 4–90° and 20–42° 2θ, step size of 0.04°, and measurement time of 2 s).

The XRD-measurement of the NCM-material is shown in Figure 1. The main peak is that of graphite, with a 30.5 wt % black mass content, according to the manufacturer. Lithium is present as lithium carbonate and lithium metal oxide. The used black mass is a mixture of various NCM-materials (e.g., Li_1.2_Co_0.1_Mn_0.556_Ni_0.13_O_2_ and Li_1.6_Co_0.2_Mn_0.3_Ni_0.3_O_2_). Lithium carbonate is included in the used black mass because of the thermal pretreatment for binder removal by the manufacturer.

The total inorganic carbon (TIC) in the leaching solution was measured using a Shimadzu TOC-L and an autosampler Shimadzu ASI-L. The samples were diluted with ultrapure water. The acidification of the sample was done with phosphoric acid (*w* = 25%).

### Experimental Procedure

Preliminary experiments were performed in a temperature controlled water shaking bath (WB, GFL 1083) for various S/L-ratios at a shaking rate of 200 rpm. 10 mL of tempered deionized water and appropriate quantities of the NCM-material were filled in test tubes (*V* = 14 mL) and sealed. These experiments were completed after 48 h. A total of 13 test tubes were prepared for each experiment. At specific times during the experiment, test tubes were removed, and the sample was analyzed.

A second series of experiments was carried out in a three-necked flask (*V* = 500 mL). The temperature adjustment was accomplished using a magnetic stirrer (Heidolph Hei-PLATE Mix ‘n’ Heat Core+), a Heat-On block, and a temperature sensor Pt-1000. The stirring of the solution was accomplished with an oval magnetic stir fish made of PTFE. To condense the evaporated liquid, a Dimroth condenser connected to a cryostat (Julabo Corio CD- 200F) was utilized. For each experiment, 200 mL of deionized water was preheated in the flask. When the target temperature was achieved, black mass was added and samples (*V* = 0.3 mL) were taken at specific time steps. Each sample was pushed through a syringe filter (mesh size 0.45 μm) to filter out any solids, diluted with HNO_3_ solution (*w* = 0.65%), and analyzed with AAS. Each experiment lasted for 3 h. The experiments were executed in a temperature range of 25–90 °C, S/L-ratios of 25–500 g L^–1^, and stirrer speeds of 200–500 rpm.

Subsequently, multistage leaching experiments were executed. These experiments were conducted with the same experimental setup as the single-stage experiments at a stirring rate of 500 rpm, S/L-ratios of 100 and 500 g L^–1^, and a temperature of 40 °C. During the experiments, samples were taken at specific time steps. Each leaching stage lasted for 1 h. The leached black mass was separated from the leaching solution by vacuum filtration after each leaching stage.

The leaching efficiency *X*_i_ and selectivity *S*_i_ (i = Li, Mn, Ni, Co, and Cu) were calculated using eqs 1 and 2, respectively.
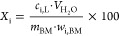
1
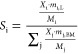
2where *c*_i,L_ is the concentration of metal i in the liquid, *V* is the volume of water, *m*_BM_ is the initial mass of black mass, *w*_i,BM_ is the mass percentage of metal i in the black mass, and *M*_i_ is the molar mass of metal i.

## Results and Discussion

### Investigation of the Operating Conditions

#### Effect of Leaching Time

**Figure 2:**
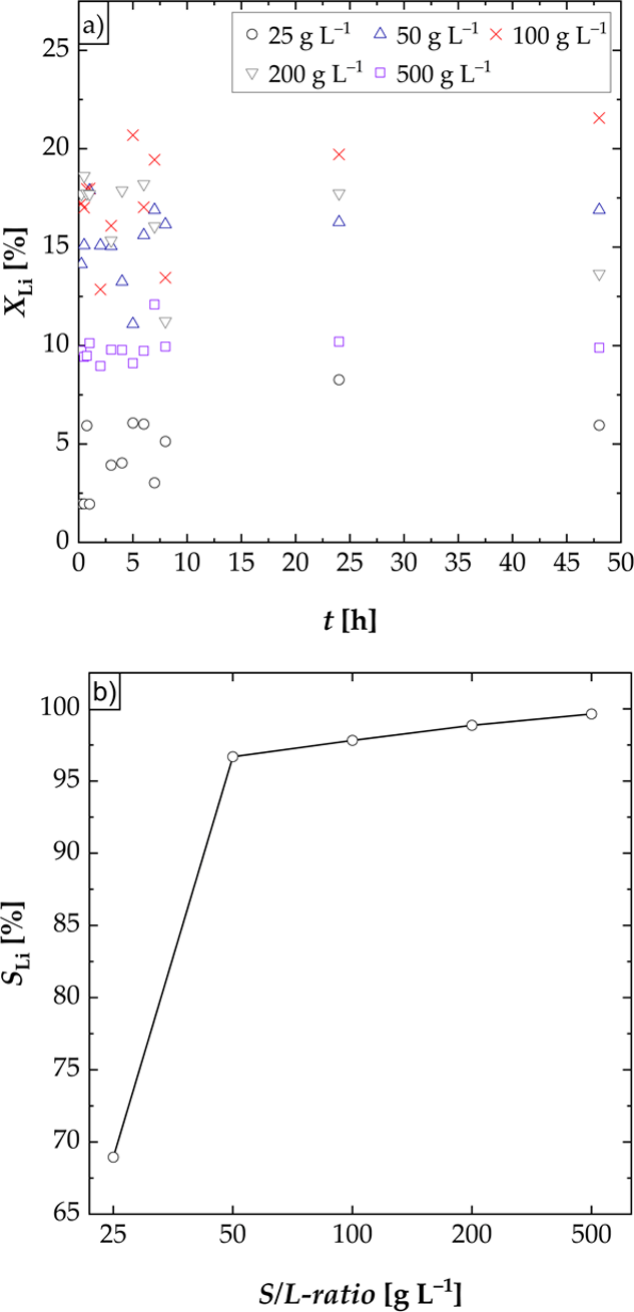
Effect of the leaching time and the solid/liquid-ratio on the leaching efficiency *X* of lithium and the selectivity *S* for lithium; experiments carried out in a water shaking bath (S/L = 25–500 g L^–1^, *n* = 200 rpm, and *T* = 60 °C); (a) leaching efficiency of Li over time; (b) selectivity for Li at *t* = 48 h.

The influence of the leaching time on the leaching efficiency of lithium was investigated in long-term experiments in a water shaking bath for 48 h. Figure 2 shows the leaching efficiencies of lithium (Figure 2a) during the long-term experiment at a temperature of 60 °C and a shaking rate of 200 rpm. The majority of lithium was leached within the first 25 min (first data point). Afterward, the concentration of lithium remained constant (Figure 2a). Figure 2a shows high scattering because the mixing efficiency in the test tubes was not high and deposits of the black mass at the bottom of the tubes were observed. Because of this, the remaining experiments were executed in a three-necked flask. The selectivity for lithium (Figure 2b) was higher than 96% for all S/L-ratios except for 25 g L^–1^ (*S*_Li_ = 68.95%).

#### Effect of Mixing

**Figure 3:**
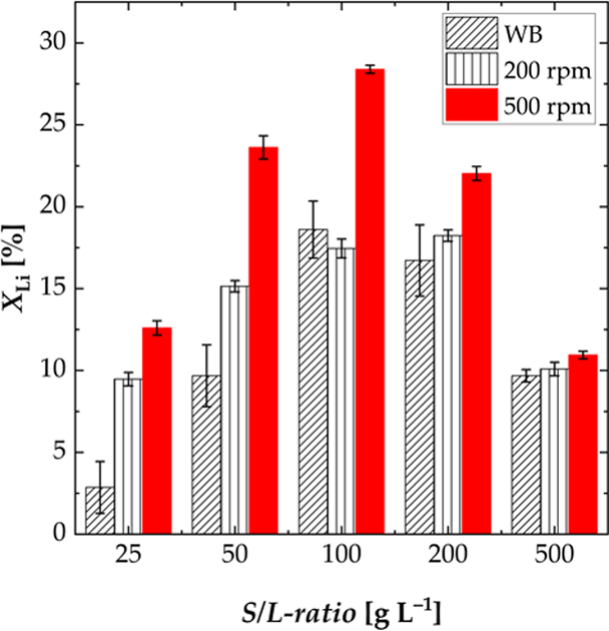
Effect of the mixing rate on the leaching efficiency *X* of lithium; experiments carried out in a water shaking bath and a three-necked flask (S/L = 25–500 g L^–1^, *n* = 200 and 500 rpm, *T* = 60 °C, and *t* = 3 h; WB: water shaking bath).

Thorough mixing is an important parameter to ensure good phase contact between black mass and water with the result of a good mass transfer. The effect of mixing on the leaching rate of lithium was thus investigated. Figure 3 shows the influence of mixing at a temperature of 60 °C and various S/L-ratios on the leaching efficiency of lithium. As expected, a higher mixing rate leads to a higher leaching efficiency which, at a S/L-ratio of 100 g L^–1^, resulted in an 11% higher leaching efficiency when stirring at 500 rpm instead of 200 rpm. In the experiments with a lower stirring speed, it was observed that a small portion of the black mass floated on the liquid and was not accessible to leaching as a result. For the higher mixing rate, the thickness of the liquid film layer surrounding the particles is less, resulting in a higher mass transfer rate.^34^ The influence of the stirring rate was scarcely visible for a S/L-ratio of 500 g L^–1^, indicating that the mass transfer was not limited by the mixing rate but by the higher slurry density.

#### Effect of S/L-Ratio

**Figure 4:**
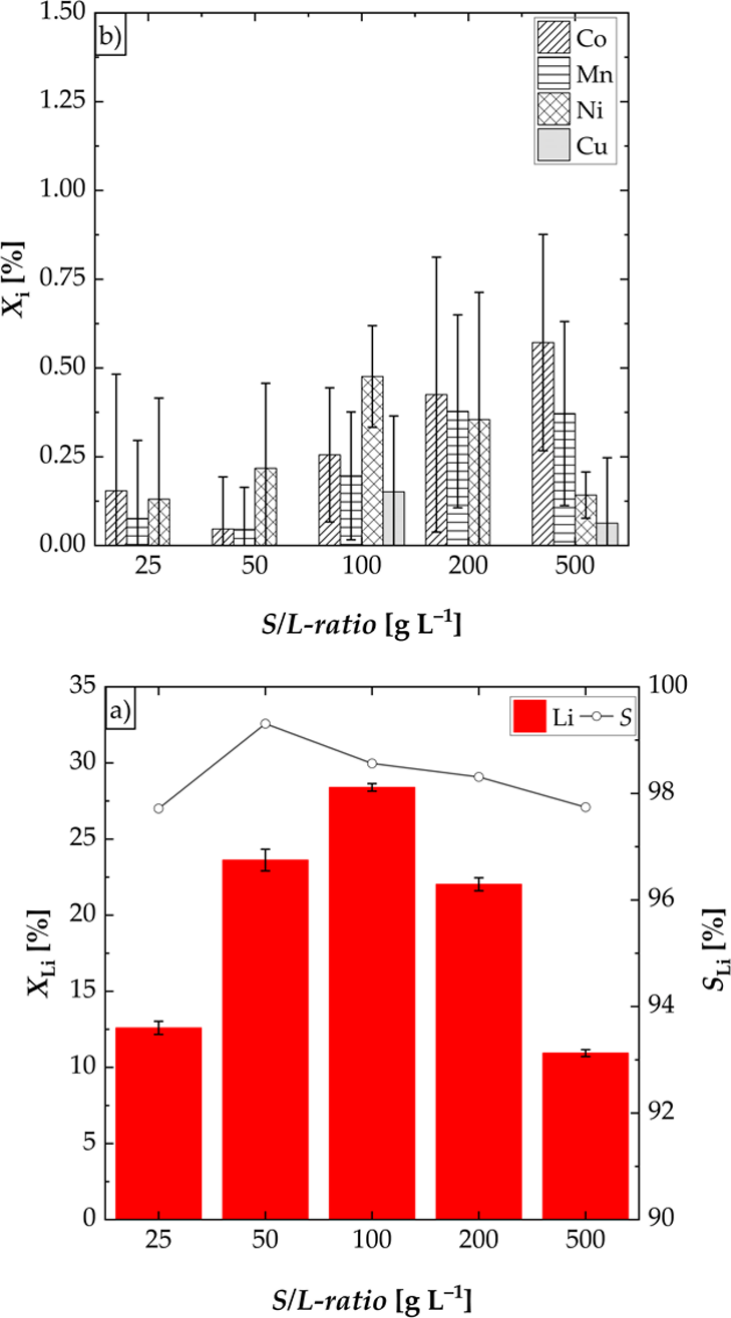
Effect of the S/L-ratio on the leaching efficiency *X* of lithium, cobalt, manganese, nickel, and copper and the selectivity *S* for lithium; experiments carried out in a three-necked flask (S/L = 25–500 g L^–1^, *n* = 500 rpm, *T* = 60 °C, and *t* = 3 h): (a) leaching efficiency of Li and selectivity for Li and (b) leaching efficiency of Co, Mn, Ni, and Cu.

To determine the optimal S/L-ratio for obtaining the maximum leaching efficiency of lithium, different S/L-ratios (25–500 g L^–1^) were investigated (Figure 4). The leaching efficiency of lithium was low, when the S/L-ratio was low (*X*_Li_ = 12.60% at S/L-ratio of 25 g L^–1^). This may be due to the fact that sufficient mixing could not be guaranteed as the black mass partially floated on a foam that was formed at low S/L-ratios and thus was not accessible to the leaching process. If the S/L-ratio was increased, a maximum leaching efficiency of lithium of 28.39% at a S/L-ratio of 100 g L^–1^ was obtained. For higher S/L-ratios, the leaching efficiency of lithium decreased again. Due to the high slurry density (200 and 500 g L^–1^), agglomeration of the black mass during leaching took place. Thus, the interfacial area between the particles of the black mass and the liquid was lowered, resulting in a lower mass transport rate. The selectivity for lithium was higher than 97% for all S/L-ratios with maximum leaching efficiencies of cobalt, manganese, nickel, and copper of 0.8% (Figure 4b).

#### Effect of Temperature

**Figure 5:**
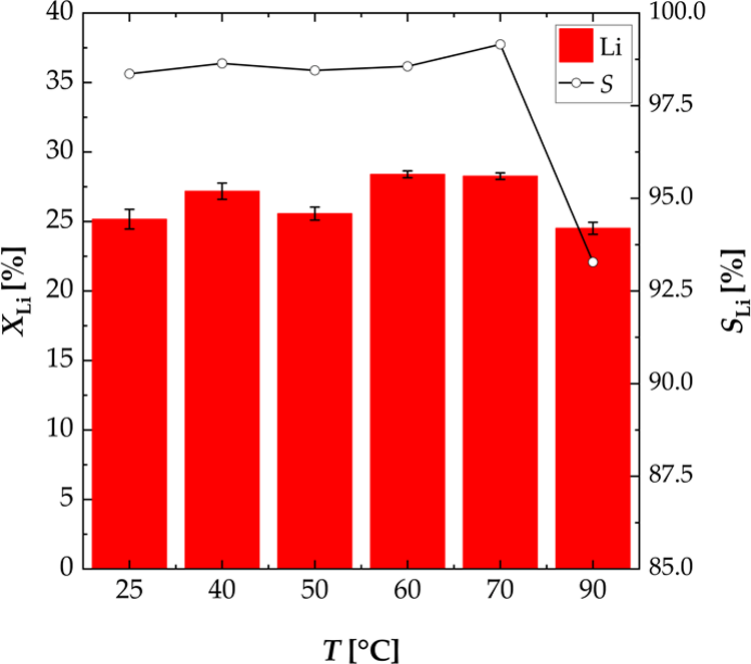
Effect of the temperature on the leaching efficiency *X* of lithium and the selectivity *S* for lithium; experiments carried out in a three-necked flask (S/L = 100 g L^–1^, *n* = 500 rpm, *T* = 25–90 °C, and *t* = 3 h).

The influence of the temperature was investigated at six different temperatures between 25 and 90 °C (Figure 5). The temperature was seen to have a minor influence on the leaching efficiency of lithium. The leaching efficiency of lithium was higher than 24.5% for all temperatures. The selectivity for lithium was higher than 98% except at 90 °C. It dropped to 93.28% at 90 °C as the efficiency for copper increased to 10.23% at that temperature. Based on these results, 40 °C was selected as the optimum leaching temperature, and consecutive experiments were carried out at 40 °C. To reduce the energy demand for heating, the multistage experiments were also carried out at a temperature of 40 °C.

**Figure 6:**
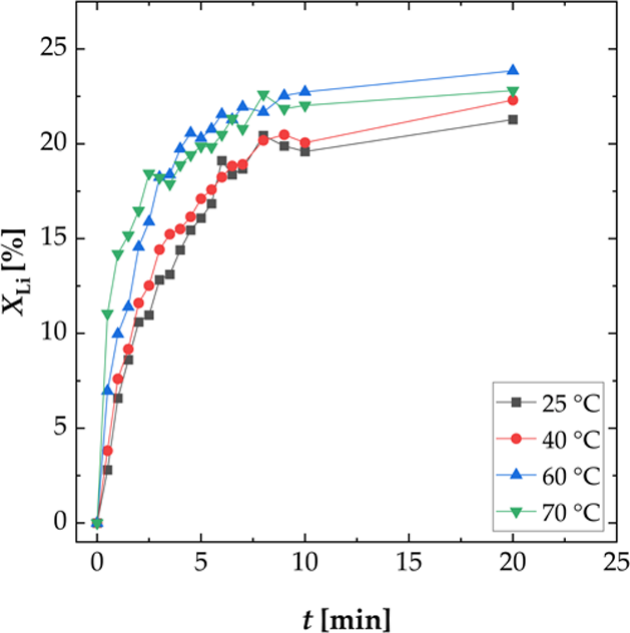
Effect of the temperature on the leaching efficiency *X* of lithium; experiments carried out in a three-necked flask (S/L = 100 g L^–1^, *n* = 500 rpm, and *T* = 25–70 °C).

The influence of the temperature on the leaching rate was determined at various temperatures from 25–70 °C (Figure 6). It was shown that the leaching rate of the two lower temperatures (25 and 40 °C) was approximately the same during the whole leaching process and was lowered to a minimum after 10 min. This indicated two leaching mechanisms. The leaching rate of the two higher temperatures (60 and 70 °C) was high at the beginning (0–3 min), decreased during the second period (3–10 min), and was subsequently lowered to a minimum after 10 min, indicating three leaching mechanisms. The modeling of the leaching kinetics is discussed in the chapter “Mechanism and kinetics”.

### Multistage Experiments

**Figure 7:**
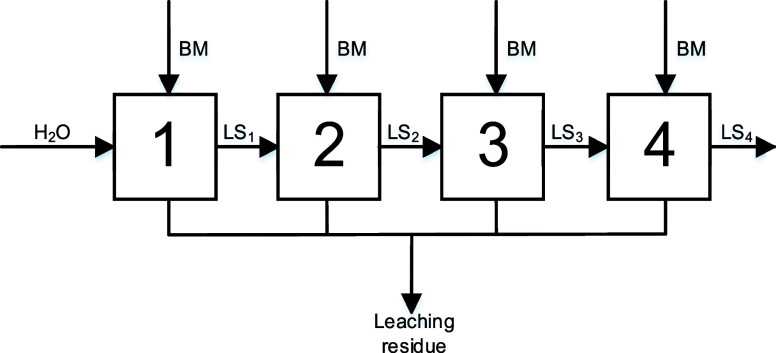
Flowsheet of the four-stage selective leaching of lithium of the NCM-material using a new black mass for every stage (S/L = 100 and 500 g L^–1^, *n* = 500 rpm, and *T* = 40 °C).

Two series of multistage experiments were carried out. First, four-stage experiments were conducted using a new black mass in every stage at S/L-ratios of 100 and 500 g L^–1^ (Figure 7).

**Figure 8:**
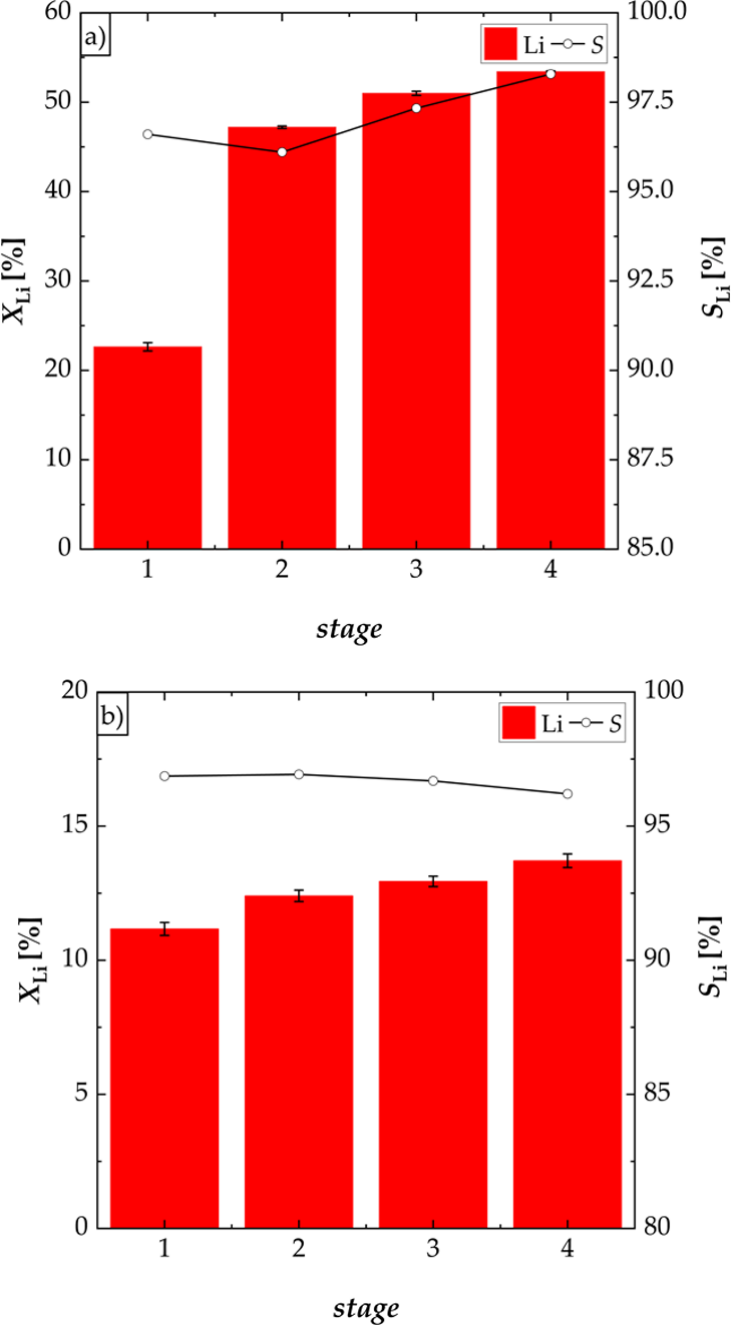
Effect of a multistage process on the leaching performance *X* of lithium and selectivity *S* for lithium using new black mass for every stage at different S/L-ratios; experiments carried out in a three-necked flask: (a) S/L = 100 g L^–1^ (four stages, *n* = 500 rpm, and *T* = 40 °C), (b) S/L = 500 g L^–1^ (four stages, *n* = 500 rpm, and *T* = 40 °C).

The leaching performance of lithium was increased from 27.18% in one stage to 53.41% (sum of efficiencies) within the four-stage process using new black mass for every stage (Figure 8a). Furthermore, the leaching efficiency of lithium was high in the first and second stage, respectively. Only minor lithium quantities were leached in the third and fourth stage. The selectivity for lithium was higher than 96% in every stage, with maximal concentrations of cobalt, manganese, nickel, and copper of 5%. The maximum concentration of lithium in the leaching solution after the fourth stage was 2.11 g L^–1^. Using a S/L-ratio of 500 g L^–1^ (Figure 8b), leaching in the first stage showed the highest leaching rate resulting in a leaching efficiency of lithium of 11.17%. After the fourth stage, the total leaching performance was increased by approximately two percent. After the fourth stage, a maximum concentration of lithium of 2.73 g L^–1^ was obtained. The selectivity for lithium was higher than 96% in every stage with maximal leaching efficiencies of cobalt, manganese, nickel, and copper of 5%.

**Figure 9:**
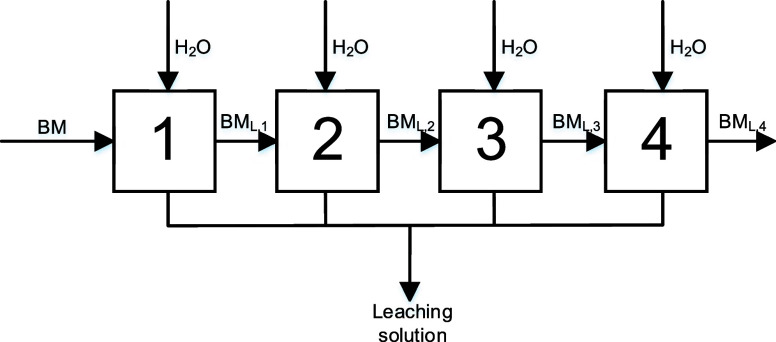
Flowsheet of the four-stage selective leaching of lithium of the NCM-material using new water for each stage (S/L = 100 g L^–1^, *n* = 500 rpm, and *T* = 40 °C).

Second, a four-stage experiment was executed using new water for every stage at a S/L-ratio of 100 g L^–1^ (Figure 9).

**Figure 10:**
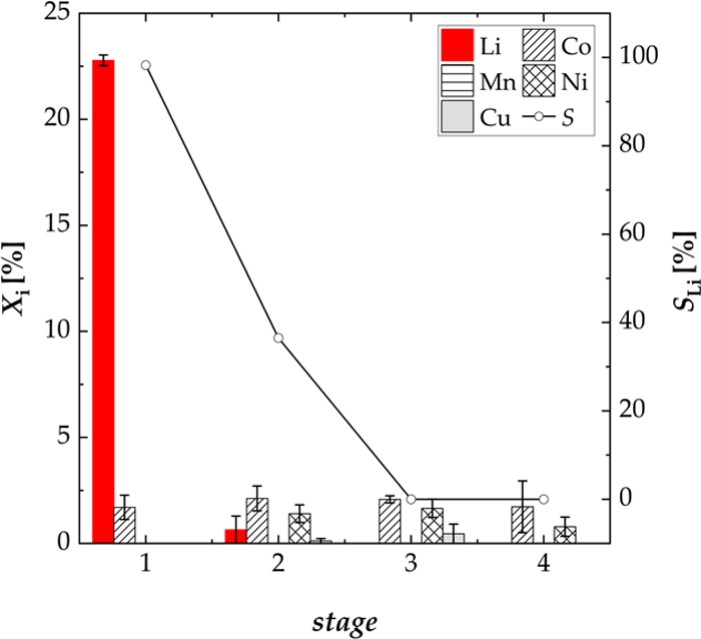
Effect of a multistage process on the leaching efficiency *X* of lithium, cobalt, manganese, nickel, and copper and the selectivity *S* for lithium using new water for every stage; experiments carried out in a three-necked flask (four stages, S/L = 100 g L^–1^, *n* = 500 rpm, and *T* = 40 °C).

Figure 10 shows the results of the four-stage process using new water for each stage. The majority of lithium was leached during the first leaching stage with a leaching efficiency of lithium of 22.78%. The selectivity for lithium was 98%. During the second stage, the leaching efficiency of lithium was lower than 1%. Furthermore, during the remaining stages (three and four), other metals (copper, nickel, and cobalt) were also leached with a maximum concentration of cobalt of 3%. Thus, it can be concluded that the entire soluble portion of lithium was leached during the first stage. Consecutive stages do not contribute to leaching of lithium but lead to leaching of the remaining metals.

**Figure 11:**
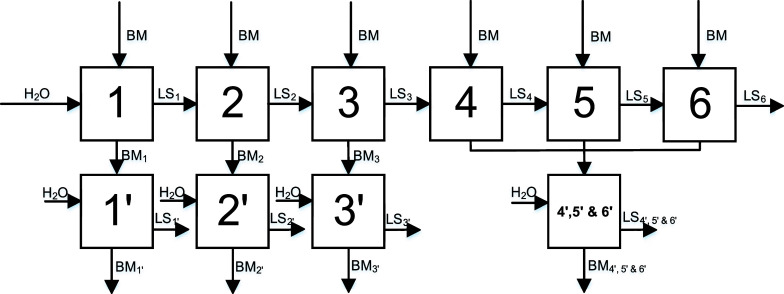
Flowsheet of the multistage leaching process of lithium from the NCM-material using new black mass for each of the stages 1–6, new water for separate stages 1′–3′, and a mixed stage 4′, 5′ and 6′ (S/L = 100 g L^–1^, *n* = 500 rpm, and *T* = 40 °C).

Finally, a setup as shown in Figure 11 was used. New black mass was utilized for each of the stages 1–6 in this experimental series. The leached black mass was then put into separate second stages 1′–3′ and a mixed stage 4′, 5′, and 6′. The experiments were carried out at a S/L-ratio of 100 g L^–1^, a mixing rate of 500 rpm, and a temperature of 40 °C.

**Figure 12:**
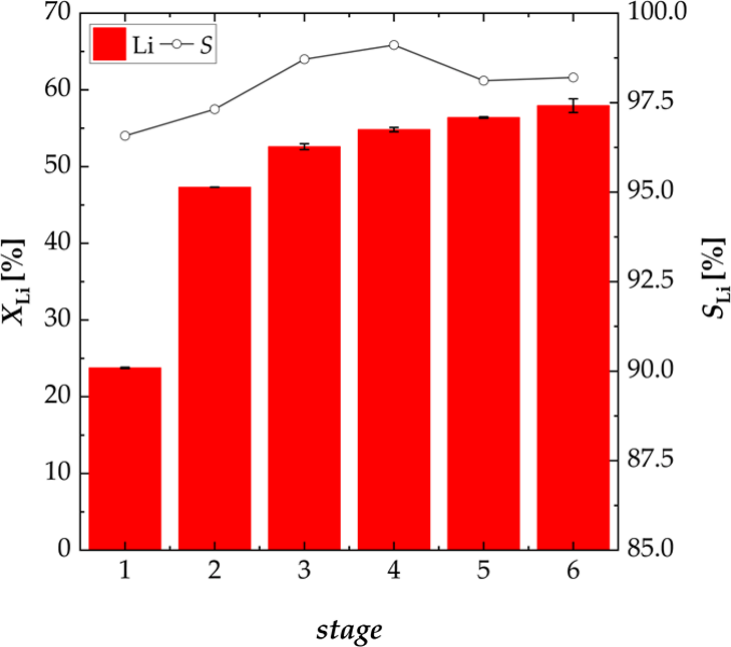
Effect of a multistage process on the leaching performance *X* of lithium and the selectivity *S* for lithium using new black mass for every stage; experiments carried out in a three-necked flask (six stages, S/L = 100 g L^–1^, *n* = 500 rpm, and *T* = 40 °C).

Figure 12 shows the results of the six-stage process using new black mass for every stage (1–6). The leaching performance at the end of the sixth stage was 57.95% with a concentration of lithium in the leaching solution of 2.27 g L^–1^. Figures S2–S5 in the Supporting Information show the efficiencies of the stages where new water was utilized. The leaching efficiency of lithium was low in stages 1′ and 2′ (*X*_Li,1′_ = 4.96% and *X*_Li,2′_ = 4.92%), as the majority of lithium had already been leached in the first stage. When less lithium was leached in the first stage, it was leached in the stages where new water was used (*X*_Li,3′_ = 15.61% and *X*_Li,4′,5′,6′_ = 15.64%), resulting in the same overall leaching efficiency of lithium. Furthermore, the leaching selectivity for lithium was strongly decreased, when black mass was leached twice (*S*_Li,1′_ = 69.09% and *S*_Li,4′,5′,6′_ = 88.93%).

**Figure 13:**
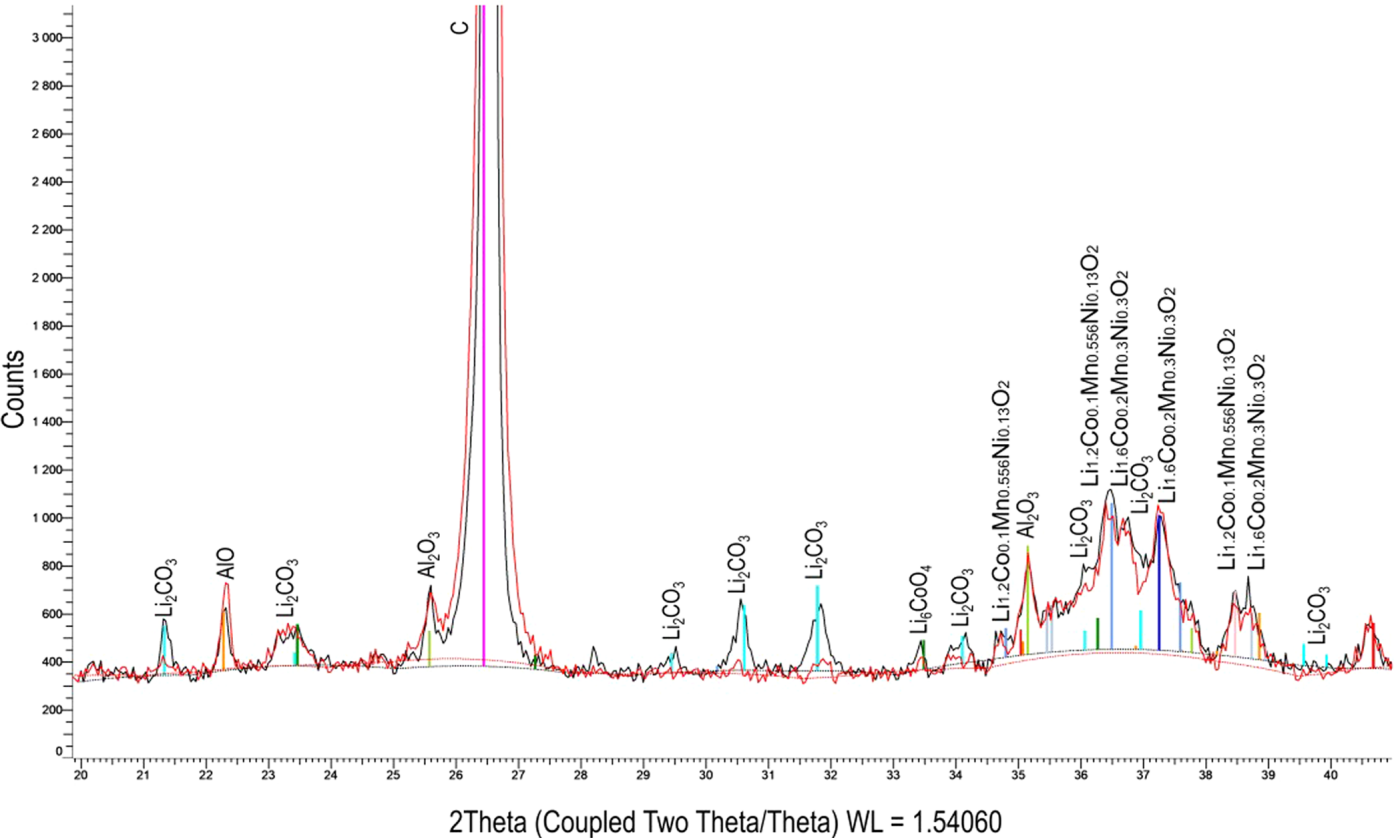
Comparison of the XRD-measurement of the NCM-material and the leached NCM-material after stage 1′ (current of 40 mA, voltage of 40 kV, scans of 4°–90° and 20°–42° 2θ, step size of 0.04°, and measurement time 2 s).

The leached black mass after stage 1′ was measured with XRD. Figure 13 shows the detailed comparison of the measurement of the NCM-material (black) and the leached NCM-material (red) after stage 1′. It was shown that the peak of lithium carbonate from the leached black mass almost vanished. Furthermore, the rest of the peaks remained constant. Therefore, it can be concluded that during the leaching, lithium carbonate was leached solely, and the concentration of the metal oxides remained constant. The concentration of lithium carbonate in the leaching solution after stage 1′ was measured with AAS and TIC. The leaching solution contained 0.85 g L^–1^ of lithium carbonate. The values obtained by AAS and TIC show a deviation of 2%, and the specified value is the mean value.

### Mechanism and Kinetics

As already described in the previous section, lithium carbonate was leached solely. Thus, it can be concluded that the leaching of lithium from black mass using water as a leaching agent is a purely physical solubility process. The solubility β of lithium carbonate with dependency of the temperature is shown in Table 2.

**Table 2:** Solubility β of Lithium Carbonate in Dependency of the Temperature^35^

*T* [°C]	0	20	25	40	50	60	70	80	90	100
β[g L^–1^]	15.4	13.3	12.8	11.5	10.7	9.9	9.2	8.5	7.8	7.2

The selective leaching of lithium from the NCM-material can be described by a kinetic model of a solid–liquid reaction.^36^ Generally, the rate is described by eq 3
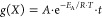
3where *g*(*X*) is the integral (reaction) model, *X* refers to the extent of the leaching process (leaching efficiency), *A* is the frequency factor, *E*_A_ is the activation energy, *R* is the universal gas constant, *T* is the temperature, and *t* is the time. Different models are available, which can be classified by the graphical shape of their isothermal curves (acceleratory, deceleratory, linear, or sigmoidal) or mechanistic assumptions (nucleation, diffusion, geometrical contraction, or reaction-order).^37,38^ The kinetics were determined for a S/L-ratio of 100 g L^–1^, a mixing rate of 500 rpm, and temperatures of 25, 40, 60, and 70 °C by a comparison of different models (Table S1 in the Supporting Information).

**Figure 14:**
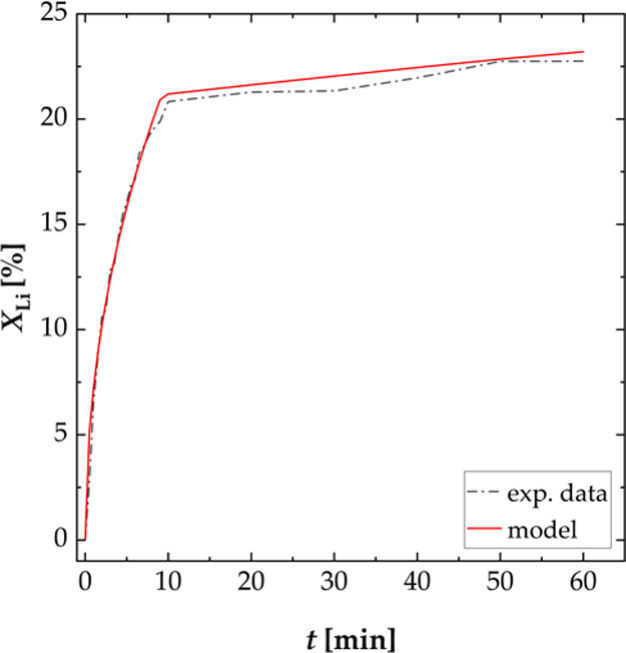
Comparison of the experimental data and the modeling for the selective leaching of lithium from the NCM-material; experiments carried out in a three-necked flask (S/L = 100 g L^–1^, *n* = 500 rpm, and *T* = 25 °C): region 0–10 min is calculated by the Jander-model for Cylindrical Diffusion, and region 10–60 min is calculated by the Interface Transfer and Diffusion Model.

When the shape of the leaching curve over time is compared (Figure 6), it can be concluded that deceleratory models result in the best description of the experimental data. For leaching temperatures of 25 and 40 °C, the leaching pathway is divided into two regions. The first region (0–10 min) is described by the Jander model for cylindrical diffusion D2 until the leaching rate reaches a minimum. The second region (10–60 min) is described by the Interface Transfer and Diffusion Model D5, where the leaching rate stays approximately constant. For leaching temperatures of 60 and 70 °C, the leaching pathway is divided into three regions. In the first region (0–3 min), the leaching rate is high and the experimental data are described by the Jander-model for Cylindrical Diffusion D2. Within the second region (3–10 min), the leaching rate is lower and the data can be described by Diffusion through Product Film Control D4. The leaching rate is almost zero within the third region (10–60 min), where the data are described best by a First-order Model O1. Figure 14 shows the experimental data in comparison with the model for a temperature of 25 °C. Only diffusion models are used to describe the leaching kinetics, which confirms the existence of a purely physical process. The values of the reaction rate constants *k* are shown in Table 3. As expected, the leaching rate was high at the beginning of the leaching process and decreased in regions 2 and 3.

**Table 3:** Reaction Rate Constants *k* and Error of Determination *R*^2^ for All Temperatures^a^^,^^b^^,^^c^^,^^d^

*t* [min]	*k* [min^–1^]				*R*^*2*^			
*T* [°C]	25	40	60	70	25	40	60	70
0–3			2.84 × 10^–3^	3.61 × 10^–3^			0.98	0.87
0–10	1.36 × 10^–3^	1.43 × 10^–3^			0.97	0.97		
3–10			1.15 × 10^–3^	1.36 × 10^–3^			0.86	0.94
10–60	1.61 × 10^–5^	1.31 × 10^–5^	3.54 × 10^–4^	5.31 × 10^–4^	0.91	0.96	0.88	0.90

a Region 0–3 min is calculated by the Jander-model for Cylindrical Diffusion for 60 and 70 °C.

b Region 0–10 min is calculated by the Jander-model for Cylindrical Diffusion for 25 and 40 °C.

c Region 3–10 min is calculated by Diffusion through Product Film Control for 60 and 70 °C.

d Region 10–60 min is calculated by Interface Transfer and Diffusion Model for 25 and 40 °C and by a First-order Model for 60 and 70 °C.

**Figure 15:**
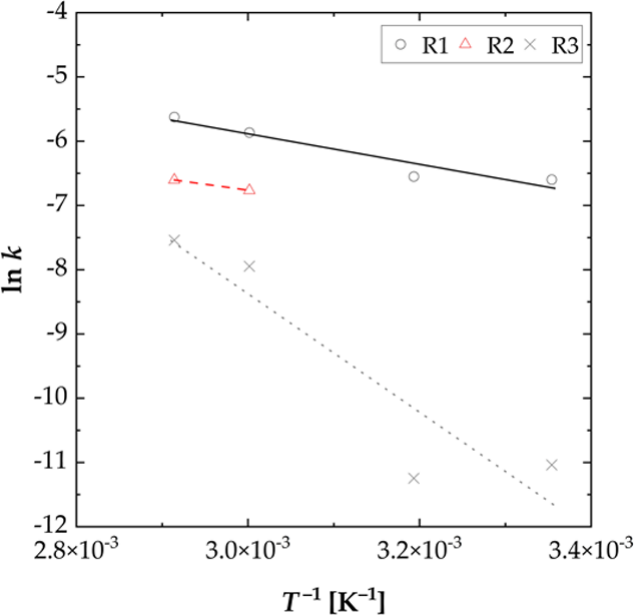
Arrhenius plot for the selective leaching of lithium from the NCM-material for the rate controlling steps (R1, 0–10 min for *T* = 25, 40 and 0–3 min for *T* = 60 and 70 °C, *R*^2^ = 0.91; R2, 3–10 min for *T* = 60 and 70 °C; R3 10–60 min for *T* = 25, 40, 60, and 70 °C, *R*^2^ = 0.85) (S/L = 100 g L^–1^, *n* = 500 rpm, *T* = 25–70 °C).

The activation energy of the leaching kinetics was calculated by the Arrhenius equation
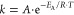
4where *A* is the frequency factor in [min^–1^], *E*_A_ is the activation energy in [J mol^–1^], *R* is the universal gas constant in [J (mol K)^−1^], and *T* is the temperature in [K]. Figure 15 shows a graphical depiction of the Arrhenius equation. The activation energies of region 1, region 2, and region 3 were determined to be 19.74, 15.43, and 76.66 kJ mol^–1^, respectively. Faraji et al.^34^ described that reactions with an activation energy less than 20 kJ mol^–1^ are diffusion controlled while activation energies higher than 40 kJ mol^–1^ suggest physical process control. The values of regions 1 and 2 confirmed that the process is diffusion controlled. Within the third region, the rate controlling step is the leaching itself.

## Conclusions

A sustainable and highly selective process for leaching of lithium from spent lithium-ion batteries (NCM-material) with water as a leaching reagent was successfully demonstrated. As optimal leaching parameters a temperature of 40 °C and a S/L-ratio of 100 g L^–1^ were identified. A leaching selectivity for lithium of higher than 98% was obtained in a single-stage experiment. The leaching efficiency of lithium was 27.18%. During multistage operation, the leaching performance of lithium increased to 57.95%. The results showed that lithium carbonate can be quantitatively leached with water without special pretreatment of the NCM-material. The advantages of water as a leaching reagent are that no further foreign ions are introduced into the system by the leaching reagent itself as well as the high selectivity for lithium, which simplifies consecutive lithium isolation via crystallization/evaporation. Kinetic modeling was performed using the experimental data from the single-stage experiments. The results showed that the first part of the leaching process (0–10 min) is diffusion controlled, and the second part (10–60 min) is controlled by the physical leaching process. Further research is intended to also address the lithium oxide fraction, which was not leached during this experimental research. One way to address the lithium oxide fraction and to enhance lithium recovery is to add a thermal pretreatment procedure (e.g., carbothermic) in combination with water as a leaching reagent for the selective leaching of lithium.

## Supporting Information Available

Particle size distribution, effect of a multistage process, and kinetic models (PDF)
