# Supplementary material for: Water as a Sustainable Leaching Agent for the Selective Leaching of Lithium from Spent Lithium-Ion Batteries
Source: ACS Omega. 2024 Feb 9;9(7):7806–16. doi: 10.1021/acsomega.3c07405 (PMC10882684; doi:10.1021/acsomega.3c07405)
Supplement: Supplementary file 1 — ao3c07405_si_001.pdf [file ao3c07405_si_001.pdf]

Water as a Sustainable Leaching Agent for the Selective Leaching of Lithium from spent Lithium-Ion Batteries

*Rafaela Greil[1], Joevy Chai[1,2], Georg Rudelstorfer[1], Stefan Mitsche[3], Susanne Lux[1]**

[1]Institute of Chemical Engineering and Environmental Technology, Graz University of Technology, NAWI Graz, Inffeldgasse 25C, 8010 Graz, Austria

[2]Chemical Engineering Department, Universiti Teknologi PETRONAS, 32610 Seri Iskandar, Malaysia

[3]Institute for Electron Microscopy and Nanoanalysis and Center for Electron Microscopy, Graz University of Technology, NAWI Graz, Steyrergasse 17, Graz 8010, Austria

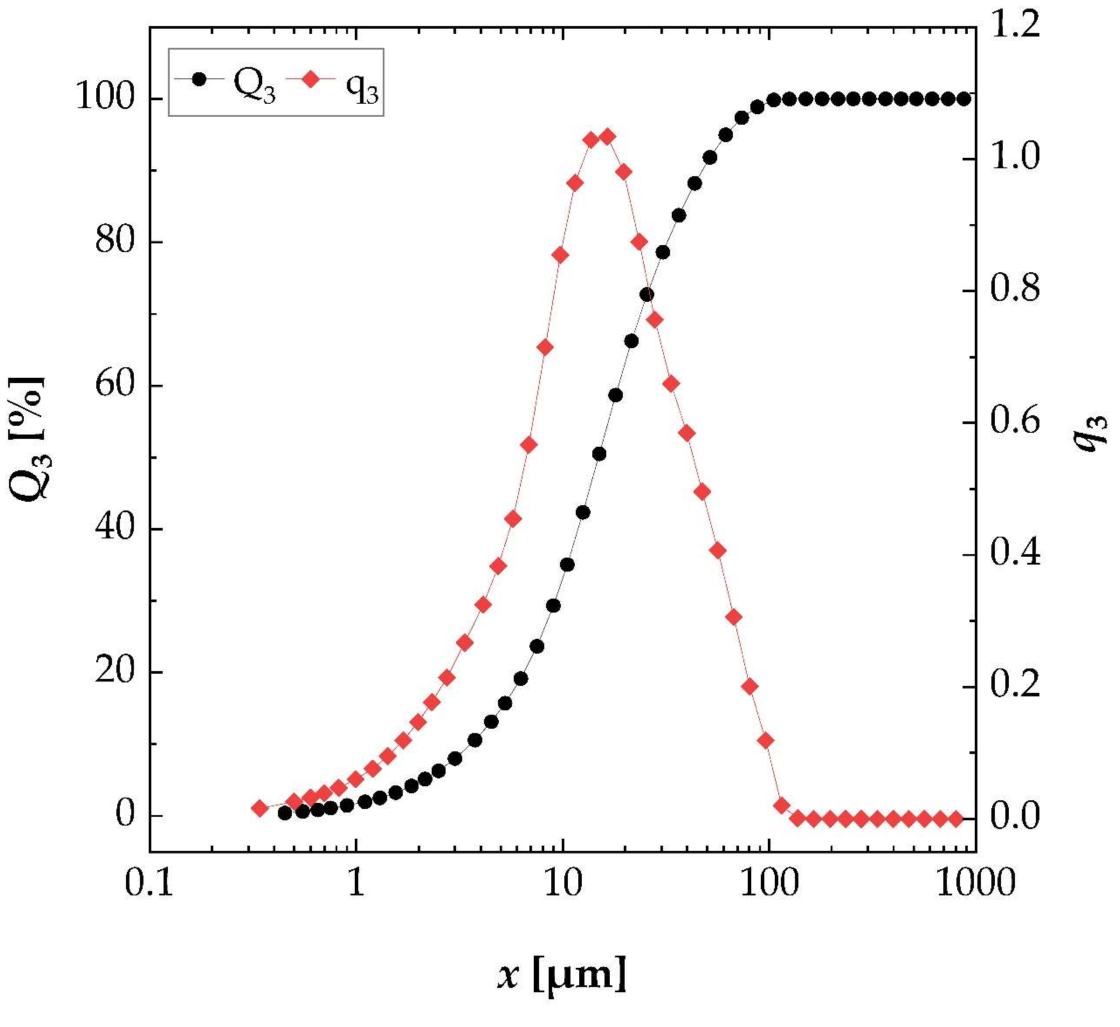

Figure S1 Particle size distribution of the NCM-material.

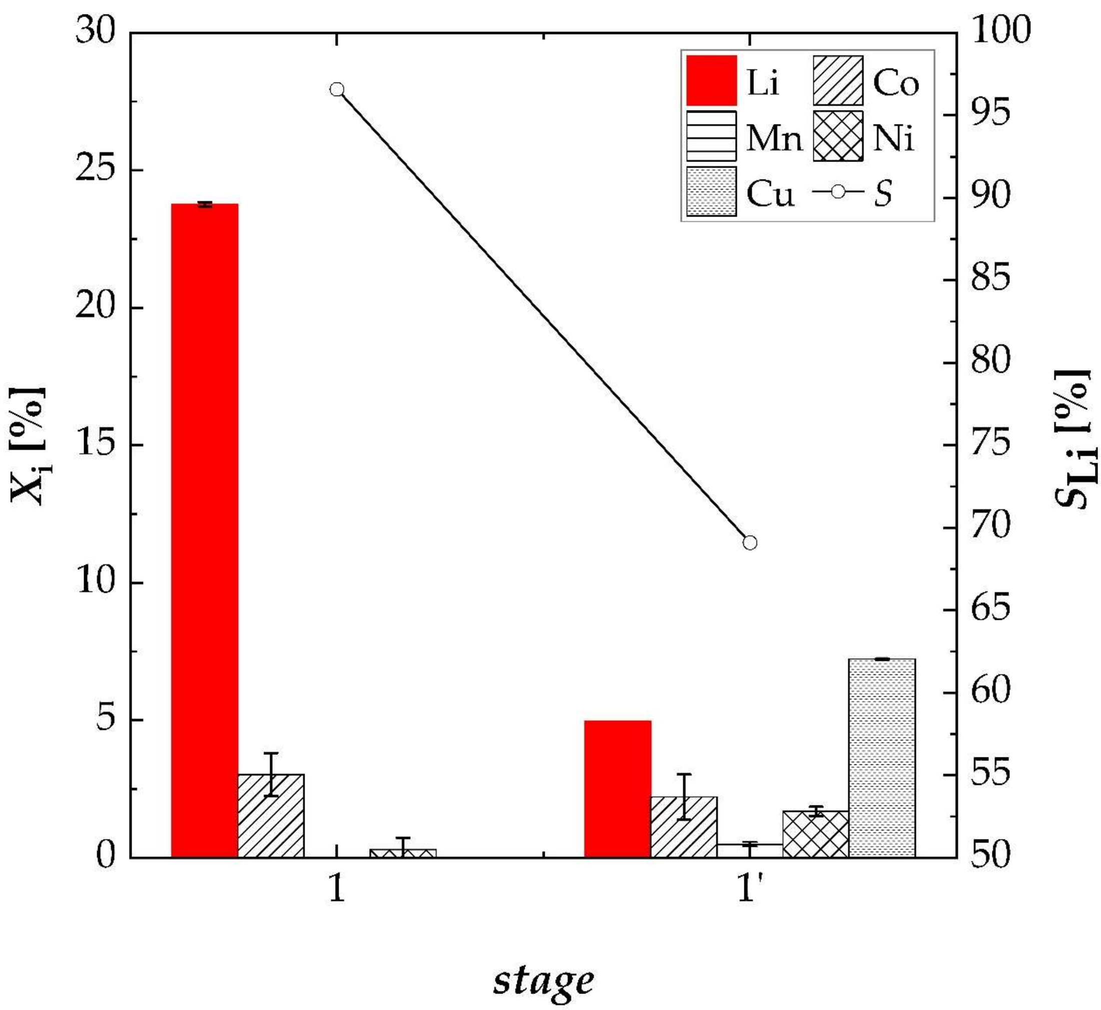

Figure S2 Effect of a multistage process on the leaching efficiency X of Li, Co, Mn, Ni and Cu and selectivity S for lithium using new water for every stage; experiments carried out in a three-necked flask (stage 1 and 1’, S/L = 100 g L^{-1}, n = 500 rpm, and T = 40 °C).

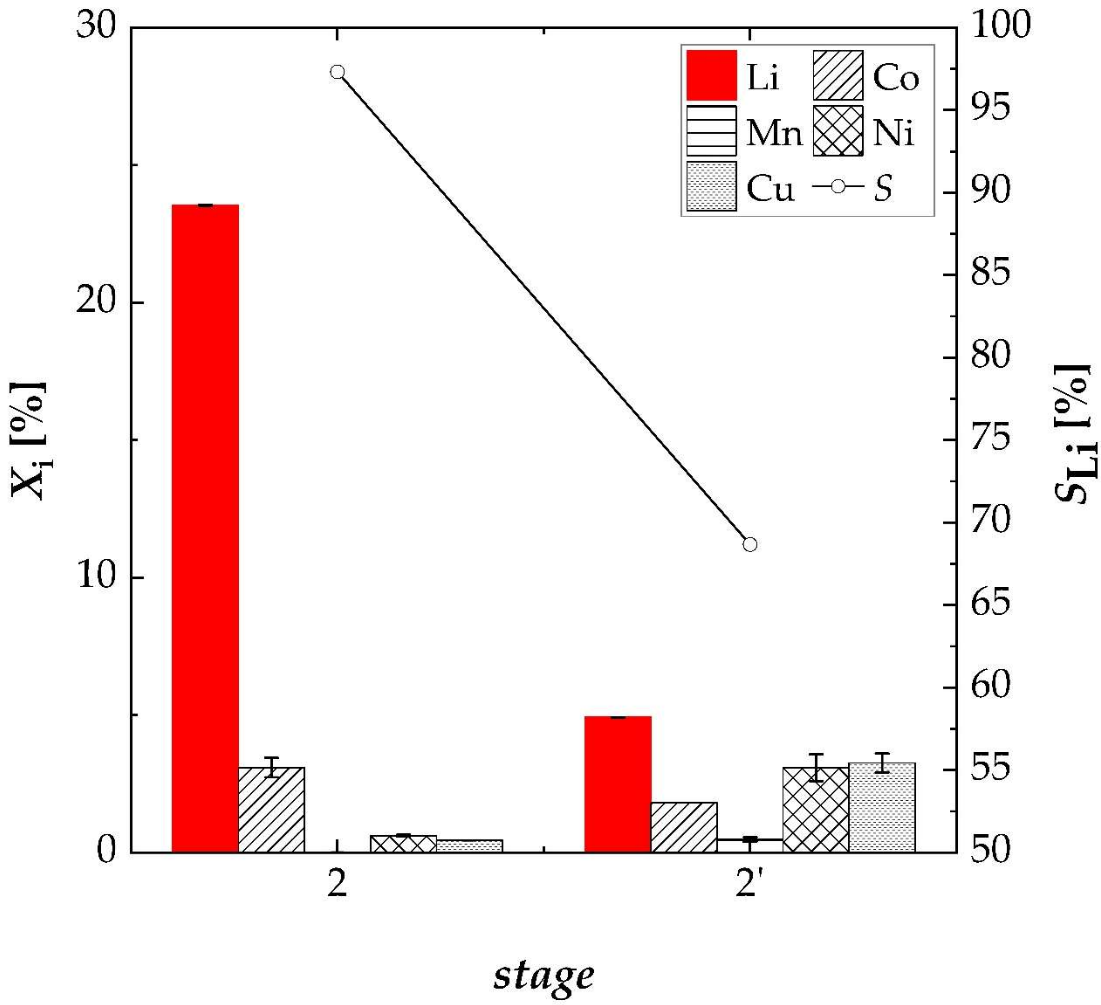

Figure S3 Effect of a multistage process on the leaching efficiency *X* of Li, Co, Mn, Ni and Cu and selectivity *S* for lithium using new water for every stage; experiments carried out in a three-necked flask (stage 2 and 2’, S/L = 100 g L^{-1}, n = 500 rpm, and T = 40 °C).

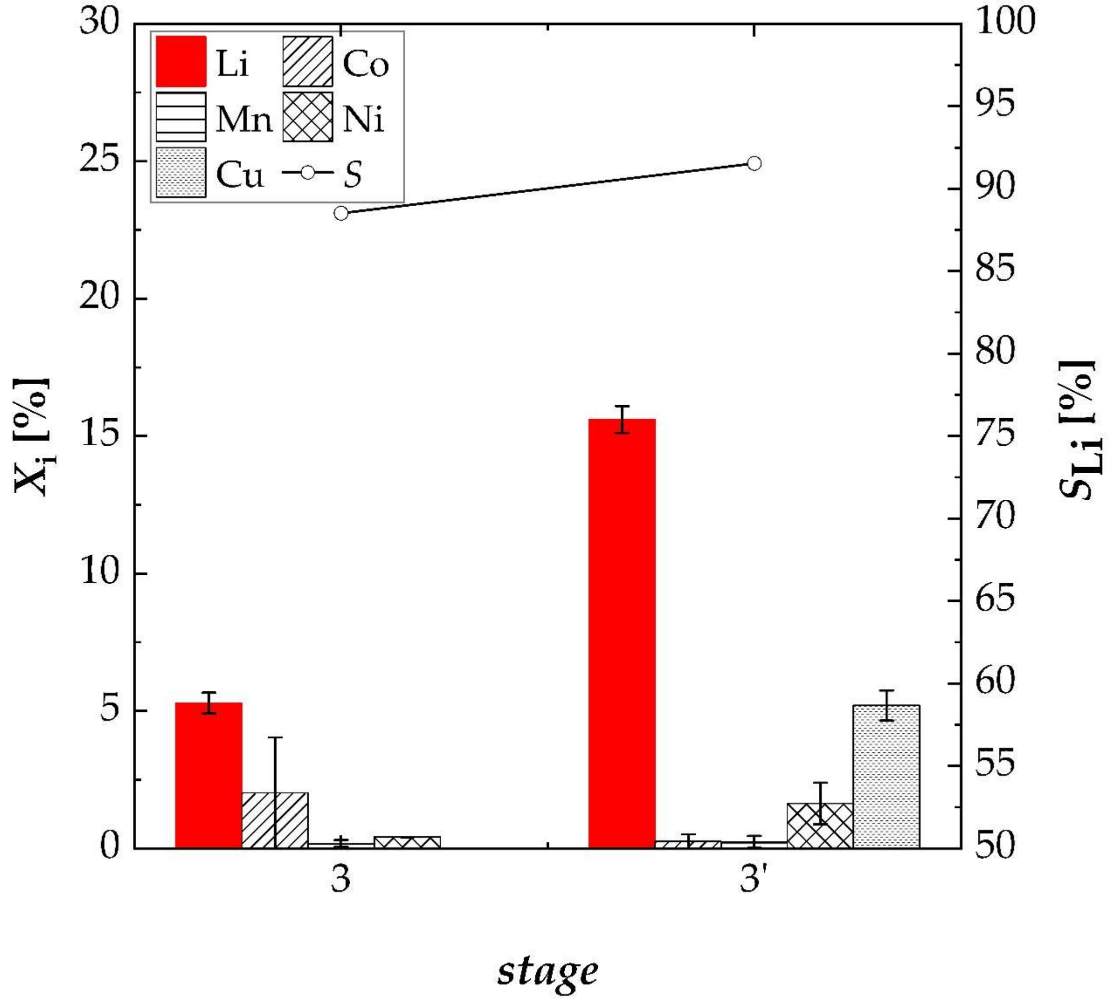

Figure S 4 Effect of a multistage process on the leaching efficiency *X* of Li, Co, Mn, Ni and Cu and selectivity *S* for lithium using new water for every stage; experiments carried out in a three-necked flask (stage 3 and 3', S/L = 100 g L^{-1}, n = 500 rpm, and T = 40 °C).

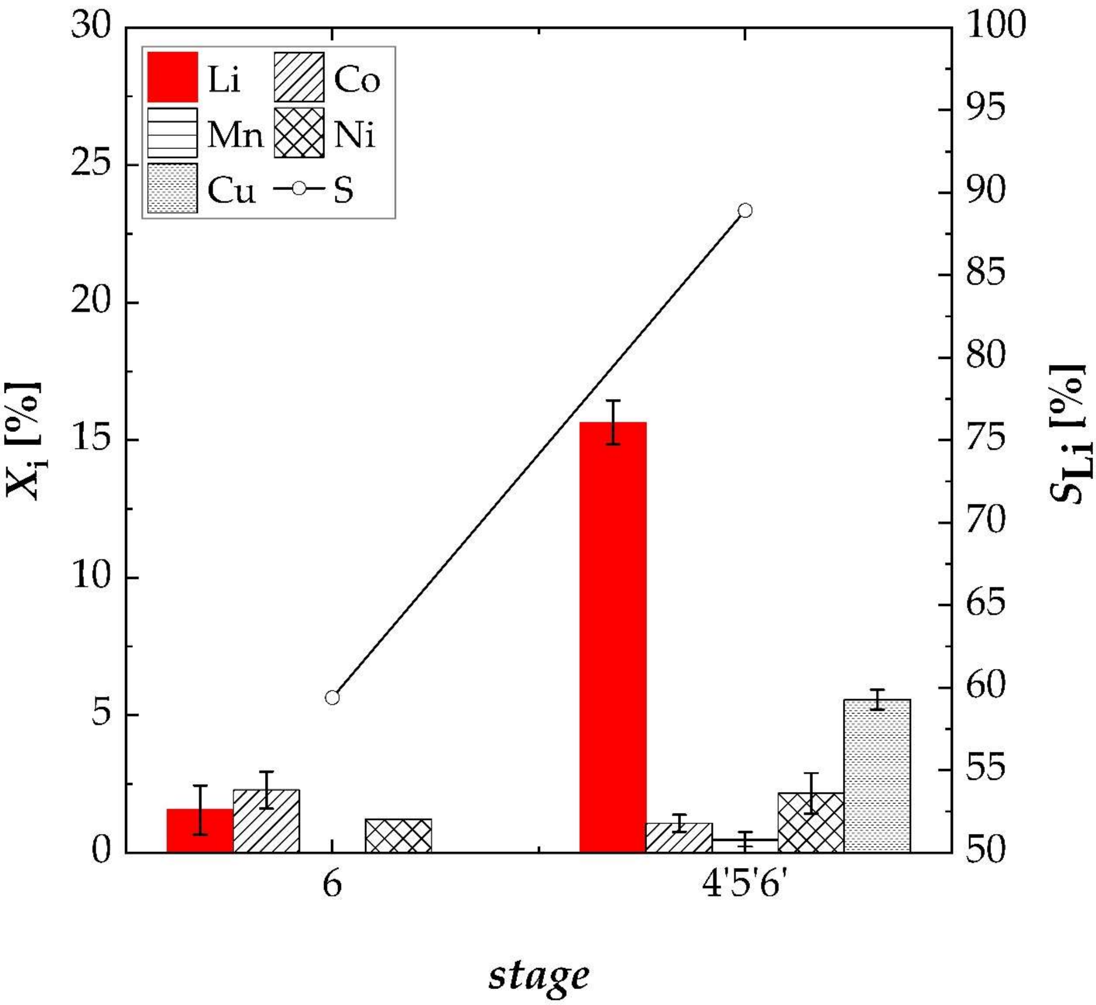

Figure S5 Effect of a multistage process on the leaching efficiency *X* of Li, Co, Mn, Ni and Cu and selectivity *S* for lithium using new water for every stage; experiments carried out in a three-necked flask (stage 6 and 4’5’6’, S/L = 100 g L^{-1}, n = 500 rpm, and T = 40 °C).

Table S1 Kinetic models for solid-liquid reactions based on their mechanistic assumptions.

#	$g(X)$	Name, description
		Nucleation
N1	$-\ln(1-X)^{\frac{1}{m}}$	Avrami, sigmoidal[33,34]
N2	$\ln\left(\frac{X}{1-X}\right)$	Prout-Tomkins[33,34]
		Diffusion
D1	$\left(1-(1-X)^{\frac{1}{3}}\right)^2$	Jander (three dimensional), deceleratory[34]
D2	$\left(1-(1-X)^{\frac{1}{2}}\right)^2$	Jander (cylindrical), deceleratory [34]
D3	$1-\frac{2}{3}X-(1-X)^{\frac{2}{3}}$	Ginstling-Brounshtein, deceleratory [33]
D4	$1-3(1-\mathrm{X})^{\frac{2}{3}}+2(1-X)$	Diffusion through Product Film Control, deceleratory [32]
D5	$\left(\frac{1}{1-X}\right)^{\frac{1}{3}}-1+\frac{1}{3}\ln(1-X)$	Interface Transfer and Diffusion Models, deceleratory [34]
D6	$\frac{1}{5}(1-X)^{-\frac{5}{3}}-0.25(1-X)^{-\frac{4}{3}}+\frac{1}{20}$	
		Geometrical contraction
G1	$1-(1-X)^{\frac{1}{2}}$	Contracting area, deceleratory
G2	$1-(1-X)^{1/3}$	Contracting volume, deceleratory
		Reaction order
O1	$-\ln(1-X)$	First-order, deceleratory
O2	$\frac{1}{1-X}-1$	Second-order, deceleratory
O3	$0.5(1-X)^{-2}-1$	Third-order, deceleratory
